# Burden of Postinfectious Symptoms after Acute Dengue, Vietnam

**DOI:** 10.3201/eid2901.220838

**Published:** 2023-01

**Authors:** Dong Thi Hoai Tam, Hannah Clapham, Elisabeth Giger, Nguyen Tan Thanh Kieu, Nguyen Tran Nam, Dinh Thi Tri Hong, Banh Thi Nuoi, Nguyen Thi Hong Cam, Nguyen Than Ha Quyen, Hugo C. Turner, Thomas Jaenisch, Cameron P. Simmons, Phung Khanh Lam, Bridget Wills

**Affiliations:** Oxford University Clinical Research Unit, Wellcome Trust Asia Programme, Ho Chi Minh City, Vietnam (D.T.H. Tam, E. Giger, N.T.T. Kieu, D.T.T. Hong, B.T. Nuoi, N.T.H. Cam, N.T.H. Quyen, P.K. Lam, B. Wills);; University of Oxford, Oxford, England, UK (H. Clapham, B. Wills);; National University of Singapore and National University Health System, Singapore (H. Clapham);; City Children’s Hospital, Ho Chi Minh City (N.T. Nam);; Imperial College London, London, England, UK (H.C. Turner);; Heidelberg University Hospital, Heidelberg, Germany (T. Jaenisch);; Colorado School of Public Health, Aurora, Colorado, USA (T. Jaenisch);; Monash University, Melbourne, Victoria, Australia (C.P. Simmons);; University of Medicine and Pharmacy at Ho Chi Minh City, Ho Chi Minh City (K. Lam)

**Keywords:** dengue, viruses, vector-borne infections, burden, Vietnam

## Abstract

We assessed predominantly pediatric patients in Vietnam with dengue and other febrile illness 3 months after acute illness. Among dengue patients, 47% reported >1 postacute symptom. Most resolved by 3 months, but alopecia and vision problems often persisted. Our findings provide additional evidence on postacute dengue burden and confirm children are affected.

Dengue is a mosquitoborne viral infection found across much of the tropical and subtropical world. Most infections are asymptomatic or paucisymptomatic. Acute symptoms range from an influenza-like self-limited febrile illness to, in a small proportion of cases, severe and complicated disease that can prove fatal (*1*). In total, 4 dengue viral serotypes (DENV-1–4) exist; severe disease rarely occurs during the first exposure to any serotype (i.e., a primary infection) but is more likely to occur during a subsequent infection with a different serotype (i.e., a secondary infection).

The symptoms of acute dengue are generally understood to resolve after 1–2 weeks, but the potential for persistent or delayed symptoms has received increasing attention in recent years. However, few formal studies have been published, and these studies have reported a range of symptoms and frequencies (*2*–*8*). A recent review summarizing this literature showed a substantial proportion of persons experienced some kind of postacute symptoms; the proportion decreased over time after infection (*9*), and 24% reported notable fatigue (*4*). 

## The Study

We report on postinfectious symptoms in 247 predominantly pediatric patients from Vietnam 3 months after an acute febrile illness; 200 of them had dengue (Appendix). After acute dengue, we observed a broad spectrum of postviral symptoms ranging from fatigue, joint pain, and muscle pain to vision problems and hair loss (Table). We report ≈8% patients experienced fatigue, consistent with a study in Singapore reporting 9% (*3*), but lower than the 24% in another Singapore study (*2*) and the 28% reported from Cuba (*4*). The Cuba study also reported headaches in 15% of patients compared with our estimate of 4%, whereas a recent study of 79 dengue-infected persons in Mexico indicated that 38% reported headaches in the second week after onset of fever, which dropped to 8% at 6–8 months (*10*). Our estimate of 47% of persons experiencing >1 symptom is higher than the 8.5% observed in Peru (*8*) but lower than the 65% experiencing >1 persistent symptom observed in Brazil (*6*). In general, the sample sizes were small in all studies, and the study methods or timeframes after infection differed.

**Table:** Number and percentage estimates of persons experiencing postacute symptoms after dengue or other febrile illness during the 3-month follow-up period, Vietnam

Symptom	Other febrile illness, n = 47			Dengue, n = 200	
	No.	% (95% CI)		No.	% (95% CI)
Alopecia	2	4.3 (0.5–14.5)		25	12.5 (8.3–17.9)
Tiredness	4	8.5 (2.4–20.4)		17	8.5 (5.0–13.3)
Resumed daily activities	47	100 (92.5–100)		200	100 (98.2–100)
Headaches	3	6.4 (1.3–17.5)		6	3.0 (1.1–6.4)
Muscle pain	0	0 (0.0–7.6)		3	1.5 (0.3–4.3)
Joint pain	2	4.3 (0.5–14.5)		3	1.5 (0.3–4.2)
Loss of appetite	2	4.3 (0.5–14.5)		3	1.5 (0.3–4.3)
Blurred vision	9	19.1 (9.2–33.3)		22	11.1 (7.0–16.2)
Rash	3	6.4 (1.3–17.5)		21	10.5 (6.6–15.6)
Sleep problem	2	4.3 (0.5–14.5)		9	4.5 (2.1–8.4)
Concentration problem	6	12.3 (4.8–25.7)		19	9.5 (5.8–14.4)
Little interest	1	2.1 (0.1–11.3)		1	0.5 (0.0–2.8)
Depressed	0	0 (0.0–7.6)		0	0 (0.0–1.8)
Other problems, including alopecia	3	6.4 (1.3–17.5)		32	16.0 (11.2–21.8)
Any symptom	22	47 (32.0–62.0)		92	46 (39.0–53.0)
Other acute illness	14	29.8 (17.3–44.9)		33	16.5 (11.6–22.4)

Symptoms have previously been associated with older age, but in our study the only symptom observed to be more likely in adults than children was joint pain (Appendix Table 2). Other studies have noted a higher frequency of symptoms in female than male patients (*2*,*5*,*6*,*8*). We noted this difference for alopecia and joint pain only; few men (3%) experienced either of these symptoms compared with ≈30% of women (Appendix Table 3). As for most other studies assessing the relationship between postinfectious symptoms and disease severity (*2*,*5*), we did not observe any relationship between symptoms after infection and disease severity during acute infection (Appendix Table 4). The numbers were small, but our study indicated worse symptoms (loss of appetite, blurred vision, and concentration problems) might be more likely after DENV-3 infection than infection with other serotypes (Appendix Table 4). This suggestion remained after controlling for disease severity and primary or secondary infection. Whether postacute symptoms vary by serotype is a possible line of future study.

The alopecia we report in our study (25/200 [13%] in dengue vs. 2/47 [4%] in other febrile illness [OFI]) has been observed previously, at a much lower rate in 1 study in Brazil (*7*) and at a similar rate in a recent study from Mexico (*10*). Alopecia after dengue has been noted in 1 case report (*11*). We identified alopecia in our study only in the category of other symptoms, and it was reported by patients without specific prompting, so this result is striking. Vision problems associated with dengue have previously been reported but mainly during the acute phase or soon afterwards (*12*,*13*). In our study, we saw that these symptoms can persist for several months or start much later after infection (Appendix Table 6), which was also seen in the recent study in Mexico (*10*). We found no association between specific symptoms during acute infection and afterwards (Appendix Table 5).

For many of the symptoms we report, occurrence rates were similar in the dengue and OFI groups (Table). Although the OFI group was relatively small and we do not have specific diagnoses for these persons, the data suggest that the late effects of dengue are not dissimilar to those experienced after other acute febrile illnesses. Our enrollment criteria and the fact that most patients recovered without additional therapy suggest a likely viral etiology; the pathogens causing disease in the control groups are likely to be quite variable between geographic locations, possibly explaining our lower rate of postacute consequences in the dengue group compared with the OFI groups in other studies (*3*,*8*).

Another potentially interesting observation was the lower rate of other illnesses experienced after the initial acute episode in the dengue group compared with the control group (33/200 [16.5%] vs. 14/47 [30%]) (Table). This lower rate might suggest some nonspecific immune modulation after dengue that is protective, or the rate in the other group could be higher than usual because of an effect of the other febrile illnesses.

## Conclusions

Understanding the burden of postacute symptoms is key to calculating the overall disease burden of dengue (*14*). A recent review estimated that the economic cost of persistent symptoms after dengue in Mexico alone was US $22.6 million (2012 prices) (*9*). In those estimates, the authors assumed symptoms were only experienced in adults because they saw an increase in the proportion of persons experiencing symptoms with age. We clearly show that children also experience postacute symptoms. In countries such as Vietnam, where much of the acute disease occurs in children, including postacute consequences in this group might change burden estimates considerably. In the Global Burden of Disease 2013 Study, 44% of the estimated total number of disability-adjusted life-years (DALYs) lost because of dengue was attributed to persistent symptoms (*15*). In recent Global Burden of Disease estimates, 8.5% of cases are assumed to experience acute consequences and are given a disability weight for chronic fatigue lasting for 6 months. In the context of our results, 8.5% might be a fairly realistic estimate; however, 100% of our patients had returned to work or normal daily life by 3 months postinfection. How accurately the infectious disease–postacute consequences disability weight currently being used represents the severity of postacute consequences is uncertain. We also observed that most symptoms lasted <1 month, suggesting 6 months is an overestimate of the duration of postacute consequences for this setting. This observation highlights the need for further research in this area because such burden calculations can influence public health priority-setting and funding decisions.

In summary, we have provided estimates of the proportion of dengue infections, mainly in children, that result in longer-term symptoms in a population in Vietnam. In addition to previously observed tiredness and joint pain, we have provided evidence for 2 longer-term symptoms, hair loss and vision problems. Further work in other settings should assess whether these symptoms are seen elsewhere. We also provide evidence that children experience long-term symptoms after dengue. This work is informative to the estimates of the burden of dengue and suggests additional information about the likely recovery path that could be given to patients when discharged after acute dengue.

Appendix Additional information about burden of postinfectious symptoms after acute dengue, Vietnam
