# Supplementary material for: Burden of Postinfectious Symptoms after Acute Dengue, Vietnam
Source: Emerg Infect Dis. 2023 Jan;29(1):160–3. doi: 10.3201/eid2901.220838 (PMC9796196; doi:10.3201/eid2901.220838)
Supplement: Appendix — Additional information about burden of postinfectious symptoms after acute dengue, Vietnam [file 22-0838-Techapp-s1.pdf]

Article DOI: https://doi.org/10.3201/eid2901.220838

Burden of Postinfectious Symptoms after Acute Dengue, Vietnam

Appendix

Persistent Symptoms after Dengue

Background Review

In one prospective study, 80% of 127 Singaporean adults experienced some degree of fatigue 2 months after discharge, with 24% reporting significant fatigue (*1*); fatigue was associated with older age, female sex, presence of chills and absence of rash during the acute illness, but not with overall acute disease severity defined in terms of dengue haemorrhagic fever (DHF) versus dengue fever (DF). However, no control group was included and relationships with the infecting DENV serotypes and/or the study participants' immune status were not assessed. Another study from Singapore reported that 9% of 118 adult dengue cases, mainly infected with DENV-1 or DENV-3, experienced persistent tiredness, drowsiness, and loss of appetite at least up to 3 weeks after the acute illness episode (*2*). The frequency of these postacute symptoms differed from a control group of patients who had experienced other febrile illnesses, with only 4% of the controls experiencing any symptoms, primarily persistent cough.

Postacute consequences have also been described in Latin America. A small study from Cuba reported that 22 of 47 adult DHF cases (47%) experienced symptoms 6 months after the acute illness, primarily weakness or lack of energy (27.6%), headache (14.8%), or arthralgia (10.6%) (*3*). Another study from Cuba reported a comparably high proportion of 57% of 97 hospitalized adults experiencing a range of symptoms in the 2 years after infection. The symptoms were not related to clinical severity, but again there was an association with female sex and some evidence for a relationship with immunological parameters (*1*). Two studies from Brazil reported symptoms over much shorter timeframes. Among 118 adult and pediatric patients 54% experienced persistent symptoms up to 1 month after acute dengue, including myalgia, weakness, hair loss, memory loss and sleepiness, again with symptoms more likely to occur in

female study participants (*4*). In the other Brazilian study, 33% of 90 adults reported some issues in the 2 months following infection (*5*), with the majority of the symptoms decreasing progressively over time after the acute illness, except that hair loss peaked 2-4 weeks after infection. However, none of the studies in Cuba or Brazil included a control group, precluding any comparisons with symptoms experienced following other febrile illnesses, or with background symptomatology in the general population.

Finally, in a large study from Peru, 7.7% of 3659 dengue patients older than 5 years reported at least one persistent symptom at a follow-up visit an average of 22 days from symptom onset, with symptom persistence progressively decreasing down to 4.3% for follow-up visits at 31-60 days; however this percentage was lower than among individuals who had experienced other febrile illnesses (10.5%) (*6*). Persistent symptoms were more likely in females and older individuals and the magnitude of these associations was greater in the dengue group compared to the control group.

Methods:

The study entitled "Clinical evaluation of dengue and identification of risk factors for severe disease" (IDAMS, NCT01550016), was a prospective multi-centre observational study that enrolled participants aged five years or more presenting within 72 hours of fever onset with symptoms consistent with possible dengue to outpatient health facilities in eight countries across Asia and Latin America (*7*). Full information on the acute clinical information collected and the laboratory diagnostics performed are described below. Hospital admission and individual case management were determined according to clinical need, and each participant was assigned an overall severity grading in line with the WHO 2009 guidelines and the recent consensus on standard clinical endpoints for dengue intervention trials (*8*,*9*).

Approximately 3 months after the acute illness episode we called sequential participants who had a) enrolled in IDAMS at one of two (of a possible three) sites in Ho Chi Minh City between September 2015 and January 2016 and b) completed the scheduled acute study visits. Up to 3 attempts were made to contact each participant. Following verbal consent for a short telephone interview, one of two specially trained staff asked questions related to their health and recovery after the acute illness episode. Participants 18 years of age and over were interviewed directly, while for those below 18 years, a parent or guardian consented to and was involved in

the interview process. The information about long term symptoms was asked according to the questionnaire documented by the telephone interviewers in a case report form (CRF) designed for the study and then double entered into an electronic database.

Binomial proportions and confidence intervals were used to compare the frequency of post-acute symptoms between individuals with confirmed dengue and those who experienced another acute febrile illness. We also summarised the time course of the various symptoms reported within the 3 month period. Among the confirmed-dengue patients for each symptom we used multivariable logistic regression to assess relationships with age (children vs adults), gender, overall acute disease severity, DENV serotype and host immune status (i.e. whether the dengue infection was primary or secondary).

For symptoms that were reported both during the acute illness and in the post-acute phase (headache, muscle pain, joint pain, loss of appetite and retro-orbital pain/blurred vision), we used logistic regression to assess potential relationships between experiencing the symptom during the acute phase and the post-acute phase.

Of 292 sequential participants enrolled at the two selected IDAMS study sites in Vietnam, all participants were eligible and 257 (88%) were contactable and provided age-appropriate consent for the telephone interview. Among this group, 200 (78%) had laboratory-confirmed dengue, 47 (18%) were diagnosed with OFI, while dengue diagnostics were inconclusive in 10.

Thus 247 individuals were included in the analysis, 186 (75%) aged 5-17 years and 61 (25%) aged 18-55 years. Dengue cases were slightly younger than non-dengue cases (mean age 14 vs 17 years). 207 of these individuals (162 dengue: 45 OFI) did not develop complications during their acute illness episode, while 25 and 2 (all with confirmed dengue) developed moderate and severe dengue respectively. Overall severity could not be classified in 13 individuals (11 dengue: 2 OFI) due to missing information. For those with dengue, serotype was determined for 196/200, with 113 (57%) infected with DENV1, 13 (7%) with DENV2, 5 (3%) with DENV3 and 65 (33%) with DENV4 (Appendix Table 1). A total of 31 infections were classified as probable primary infections, 141 as probable secondary and the remainder as indeterminate (Appendix Table 1). Among the 247 study participants there were no reported mental health problems before the suspected dengue episode. All individuals had resumed

normal activity at the time the questionnaire was completed, and no one reported feeling down, depressed or hopeless at this time.

Detailed Information on the Original IDAMS Study

1. IDAMS Study (IDAMS, NCT01550016) - participant enrollment

Children and adults presenting to outpatient facilities at designated study sites in 8 countries across Latin America and Southeast Asia, with symptoms consistent with dengue, were screened for potential study enrolment.

Inclusion criteria

• Age ≥5 years

• Fever or history of fever for ≤72 hours

• Clinical symptoms consistent with possible dengue (suspected dengue and/or undifferentiated fever in a patient from a dengue-endemic area)

• Suitable for outpatient care (i.e. no signs of severe disease) at the time of study enrolment, at the discretion of the treating physician

• Written informed consent

Exclusion criteria

• Localizing signs or symptoms suggesting an alternative diagnosis

• Unlikely to attend daily follow-up (e.g., due to travelling distance from the clinic), as judged by the treating physician

2. Assessments and clinical management

Following informed consent, a detailed clinical assessment was recorded in a case report form (CRF) at study enrolment and then daily until the individual had fully recovered, or for up to 6 days from enrolment. All patients were also asked to attend a follow-up visit between illness days 10-14, at which time a final clinical summary of the illness episode was completed. Individuals who did not attend the follow-up visit were contacted by telephone and asked to provide summary information of their progress since the final acute illness visit.

Management decisions throughout the acute illness were at the discretion of the responsible physicians, independent of study staff. Any patient requiring hospital admission continued to be followed daily by study staff up to study day 6.

3. Dengue diagnostic testing and definitions

Dengue diagnostic testing included an in-house serotype specific DENV RT-PCR, NS1 antigen detection (Platelia NS1, Biorad) and DENV IgM and IgG Capture ELISAs (Panbio, Australia), performed in accordance with established methodology and/or the manufacturers' instructions. Tests were performed in batches at the OUCRU laboratory in HCMC. NS1 detection and viral RT-PCR were attempted on all enrolment plasma samples, while serology was performed on paired plasma samples.

Confirmed dengue

Participants were classified as having confirmed dengue if positive on either RT-PCR or NS1 testing on the enrolment sample. Given the potential for cross-reactivity with other locally circulating flaviviruses such as Zika and JEV, serological responses were not considered for the confirmed dengue group.

Participants who were negative for both RT-PCR and NS1 were classified as not dengue (i.e. other febrile illness, OFI) if both the IgG and IgM serology were negative on paired samples, with the second sample obtained >= 6 days after illness onset and >2 days after the first sample.

In all other circumstances the diagnostics were considered inconclusive.

Classification of immune status

• Probable primary infection: negative or equivocal dengue-specific IgG results on two consecutive specimens taken at least 2 days apart, with at least one specimen obtained in the convalescent phase (illness days 6-10). Of note, patients without an IgG result in the convalescent phase but with a negative or equivocal IgG after day 10 were considered to have had a negative or equivocal IgG between illness days 6-10.

• Probable secondary infection: positive dengue-specific IgG identified in the early phase (illness days 1-5) and/or convalescent phase (illness days 6-10).

• Inconclusive immune status: All other cases, due to the absence of suitable specimens at appropriate time-points.

4. Definitions for clinical outcomes

Each individual was given a final severity classification using all available data. We required a minimum dataset in order to derive a meaningful clinical outcome. In addition to enrolment data, we considered either of the following scenarios as providing adequate information:

• at least 3 acute illness visits during illness days 4-7 (critical phase for complications),

• at least 1 acute illness visit during illness days 4-7 (critical phase for complications) together with an informative final follow-up visit summary.

Patients were classified into one of three groups according to the presence of evidence for vascular leakage, bleeding and/or organ impairment, in line with the WHO 2009 guidelines and the recent consensus on standard clinical endpoints for dengue intervention trials.

• Uncomplicated Dengue (Severity 1)

• Moderate Dengue (Severity 2)

• Severe Dengue (Severity 3)

Each component is defined below.

Definitions for All Components of the Clinical Outcome Classification System

Component	Definition
Plasma leakage	
Severe	Clinical shock and/or respiratory distress due to plasma leakage noted in the CRF by the study doctor
Moderate	Did not fulfill criteria for severe AND there was evidence of haemoconcentration* ≥ 20% and/or clinical or radiological evidence of fluid accumulation
Uncomplicated (neither moderate nor severe)	Did not fulfill criteria for severe or moderate plasma leakage AND intravenous fluid was not given for any of the following reasons - rehydration, bleeding, rising haematocrit - AND patient was afebrile at the last acute illness visit
Undetermined	All other situations
Bleeding	
Severe	Received packed red cells and/or whole blood during the acute illness for any reason except known pre-existing anaemia (with a consistent enrolment haemoglobin value), and/or occurrence of specific rare complications such as bleeding into a critical organ (brain, spinal cord)
Moderate	Severe bleeding noted in the CRF by study doctor but no supporting evidence for the severe criteria noted above, and/or the patient received blood products such as FFP, cryoprecipitate, or platelets but NOT packed red cells or whole blood, and/or the patient required an intervention for bleeding (eg nasal packing), and/or the patient received packed red cells/whole blood but had pre-existing anaemia according to past medical history plus a consistent haemoglobin value
Uncomplicated (neither moderate nor severe)	All other situations
Neurological involvement	

Severe	Any neurological signs/symptoms in a patient who was admitted, or occurrence of a convulsion accompanied by any other neurological signs/symptoms even if the patient was not hospitalized
Moderate	Single convulsion without other neurological signs/symptoms or need for hospitalization
Uncomplicated (neither moderate nor severe)	All other situations
Liver involvement	
Severe	Visible jaundice and/or coagulopathy and/or encephalopathy
Moderate	ALT >= 400 IU/L and/or AST >= 400 IU/L
Uncomplicated (neither moderate nor severe)	ALT and/or AST available and all values < 400 IU/L
Undetermined	No ALT or AST values available between enrolment and illness day 10
Other major organ failure	
Severe	CK abnormalities (or other enzymes if available [eg troponin]) AND functional abnormalities (reduced EF <50% or new ECG abnormalities) and/or specific intervention needed (eg inotropic support)
Moderate	Troponin and/or CK abnormalities AND a specific diagnostic intervention (eg cardiac ultrasound, cardiac MRI) but without EF <50%
Uncomplicated (neither moderate nor severe)	All other situations

*Haemoconcentration = (peak haematocrit – baseline haematocrit)/baseline haematocrit, where peak haematocrit is defined as the maximum documented haematocrit between illness day 4-7 and baseline haematocrit is defined as the minimum documented haematocrit between illness days 1-3.

Questionnaire for Adults

IDAMS – POSTVIRAL FATIGUE – Adult (18 or older)

Study code: [2 | 2 | D | X] - [|] - [| | |] Initials: [__|__|__|__|__|__|__]

BEFORE/AFTER THE ACUTE FEBRILE ILLNESS

Before the acute febrile illness (e.g. dengue), were you healthy* / well in general? ○ YES ○ NO ○ I do not remember

Before the acute febrile illness, did you ever have mental health problems (feeling down, depressed, hopeless, anxiety, severe mood disorder)? ○ YES ○ NO ○ I do not remember. If Yes, please describe the problem.

In the time since the febrile illness have you had any other acute illness or medical problem? ○ YES ○ NO. If yes, please give details (diagnosis, treatment or hopstialization required etc).

PERSISTING PROBLEMS AFTER THE ACUTE FEBRILE ILLNESS (such as DENGUE)

**HEALTH PROBLEM**	**DURATION**			
If YES, please continue to the right and estimate the duration of the persisting problem. If NO, please go directly to the next question.	<1 week	>1 week and <1 mth	> 1 month and <3 mths	>3 mths
After the acute febrile illness, were you tired easily or did you feel without any energy? ○ YES ○ NO ○ I do not remember				
After the acute febrile illness, were you able to resume the same "normal" daily activities compared to before the illness episode? ○ YES ○ NO ○ I do not remember				
**PERSISTENT SYMPTOMS**				
Persistent headaches ○ YES ○ NO ○ I do not remember				
Muscle pain ○ YES ○ NO ○ I do not remember				
Arthralgia / joint pain ○ YES ○ NO ○ I do not remember				
Loss of appetite ○ YES ○ NO ○ I do not remember				
Blurred vision / problems with eye sight ○ YES ○ NO ○ I do not remember				
Rashes / skin problems ○ YES ○ NO ○ I do not remember				
Trouble falling or staying asleep, or sleeping too much ○ YES ○ NO ○ I do not remember				
Problems with concentration ○ YES ○ NO ○ I do not remember				
Little interest of pleasure in doing things ○ YES ○ NO ○ I do not remember				
Feeling down, depressed or hopeless ○ YES ○ NO ○ I do not remember				
OTHER – please describe				

Name & signature of person completing form: [________________________________][___|___|___|___]; Date form completed: __ __ / __ __ / __ __

Questionnaire for Children

IDAMS – POSTVIRAL FATIGUE – Child

Study code: [2 | 2 | D | X] - [|] - [| | |] Initials: [__|__|__|__|__|__|__]

PERSON RESPONDING TO THE QUESTIONS

What is the relationship of the responsible adult to the child? ○ Parent ○ Grandparent ○ Relative ○ Legal Guardian ○ Other

Who responded verbally to the questions about the child? ○ The child / adolescent replied via the responsible adult ○ The child responded to the questions from the interviewer directly

BEFORE/AFTER THE ACUTE FEBRILE ILLNESS

Before the acute febrile illness (e.g. dengue), were you healthy* / well in general? (Substitute "your child" if the respondent is the responsible adult rather than the child.) ○ YES ○ NO ○ I do not remember

Before the acute febrile illness, did you ever have mental health problems (feeling down, depressed, hopeless, anxiety, severe mood disorder)? ○ YES ○ NO ○ I do not remember. If Yes, please describe the problem.
In the time since the febrile illness have you had any other acute illness or medical problem? ○ YES ○ NO. If yes, please give details (diagnosis, treatment or hopstialisation required etc).

PERSISTING PROBLEMS AFTER THE ACUTE FEBRILE ILLNESS (such as DENGUE)				
**HEALTH PROBLEM**	**DURATION**			
If YES, please continue to the right and estimate the duration of the persisting problem. If NO, please go directly to the next question.	<1 week	>1 week and <1 mth	> 1 month and <3 mths	>3 months
After the acute febrile illness, were you tired easily or did you feel without any energy? ○ YES ○ NO ○ I do not remember				
After the acute febrile illness, were you able to resume the same "normal" daily activities compared to before the illness episode? ○ YES ○ NO ○ I do not remember				
**PERSISTENT SYMPTOMS**				
Persistent headaches ○ YES ○ NO ○ Do not remember				
Muscle pain ○ YES ○ NO ○ Do not remember				
Arthralgia / joint pain ○ YES ○ NO ○ Do not remember				
Loss of appetite ○ YES ○ NO ○ Do not remember				
Blurred vision / problems with eye sight ○ YES ○ NO ○ Do not remember				
Rashes / skin problems ○ YES ○ NO ○ Do not remember				
Trouble falling or staying asleep, or sleeping too much ○ YES ○ NO ○ Do not remember				
Problems with concentration ○ YES ○ NO ○ Do not remember				
Little interest or pleasure in doing things ○ YES ○ NO ○ Do not remember				
Feeling down, depressed or hopeless ○ YES ○ NO ○ Do not remember				
OTHER – please describe				

Name & signature of person completing form: [________________________________][___|___|___|___] Date form completed: __ __ / __ __ / __ __

References

1. González D, Martínez R, Castro O, Serrano, T, Portela D, Vazquez S, et al. Evaluation of some clinical, humoral and imagenological parameters in patients of dengue haemorrhagic fever six months after acute illness. Dengue Bull. 2005;29:53–7.

2. Seet RCS, Quek AML, Lim ECH. Post-infectious fatigue syndrome in dengue infection. J Clin Virol. 2007;38:1–6. PubMed https://doi.org/10.1016/j.jcv.2006.10.011

3. Low JGH, Ooi E-E, Tolfvenstam T, Leo YS, Hibberd ML, Ng LC, et al. Early dengue infection and outcome study (EDEN)—study design and preliminary findings. Ann Acad Med Singap. 2006;35:783–9. PubMed

4. García G, González N, Pérez AB, Sierra B, Aguirre E, Rizo D, et al. Long-term persistence of clinical symptoms in dengue-infected persons and its association with immunological disorders. Int J Infect Dis. 2011;15:e38–43. PubMed https://doi.org/10.1016/j.ijid.2010.09.008

5. Teixeira LA, Lopes JSM, Martins AG, Campos FA, Miranzi SS, Nascentes GA. Persistence of dengue symptoms in patients in Uberaba, Minas Gerais State, Brazil [in Portuguese]. Cad Saude Publica. 2010;26:624–30. PubMed https://doi.org/10.1590/S0102-311X2010000300019

6. Tristão-Sá R, Kubelka CF, Zandonade E, Zagne SM, Rocha NS, Zagne LO, et al. Clinical and hepatic evaluation in adult dengue patients: a prospective two-month cohort study. Rev Soc Bras Med Trop. 2012;45:675–81. PubMed https://doi.org/10.1590/S0037-86822012000600004

7. Halsey ES, Williams M, Laguna-Torres VA, Vilcarromero S, Ocaña V, Kochel TJ, et al. Occurrence and correlates of symptom persistence following acute dengue fever in Peru. Am J Trop Med Hyg. 2014;90:449–56. PubMed https://doi.org/10.4269/ajtmh.13-0544

8. Tiga DC, Undurraga EA, Ramos-Castañeda J, Martínez-Vega RA, Tschampl CA, Shepard DS. Persistent symptoms of dengue: estimates of the incremental disease and economic burden in Mexico. Am J Trop Med Hyg. 2016;94:1085–9. PubMed https://doi.org/10.4269/ajtmh.15-0896

9. Tiga-Loza DC, Martínez-Vega RA, Undurraga EA, Tschampl CA, Shepard DS, Ramos-Castañeda J. Persistence of symptoms in dengue patients: a clinical cohort study. Trans R Soc Trop Med Hyg. 2020;114:355–64. PubMed https://doi.org/10.1093/trstmh/traa007

Appendix Table 1. Serotype and immune status for 196/200 acute dengue infections for which a serotype was identified

Primary / secondary determination	Serotype
Probable primary	DENV 1: 25
	DENV 2: 2
	DENV 3: 1
	DENV 4: 1
Probable secondary	DENV 1: 71<br>DENV 2: 8<br>DENV 3: 3<br>DENV 4: 58
Inconclusive	DENV 1: 17<br>DENV 2: 3<br>DENV 3: 1<br>DENV 4: 6

Appendix Table 2. Estimates of the number and percentage of persons experiencing each symptom in adults versus children, with 95% binomial CIs, among the confirmed dengue cases

Symptoms	No. adults, n = 45	% (95% CI)	No. children, n = 155	% (95% CI)
Alopecia	3	6.7 (1.4–18.3)	22	14.2 (9.1–20.7)
Tiredness	2	4.4 (0.5–15.1)	15	9.7 (5.5–15.5)
Resumed daily activities	45	100 (92.1–100)	155	100 (97.6–100)
Headaches	3	6.7 (1.4–18.3)	3	1.9 (0.4–5.6)
Muscle pain	0	0.0 (0.0–7.87)	3	1.9 (0.4–5.6)
Joint pain	2	4.4 (0.5–15.1)	1	0.6 (0.0–3.5)
Loss of appetite	0	0.0 (0.0–7.9)	3	1.9 (0.4–5.6)
Blurred vision	3	6.7 (1.40–18.3)	19	12.3 (7.5–18.5)
Rash	7	15.6 (6.5–29.5)	14	9.0 (5.0–14.7)
Sleep problem	4	8.9 (2.5–21.2)	5	3.2 (1.1–7.4)
Concentration problem	2	4.4 (0.5–15.1)	9	11.0 (6.5–17.0)
Little interest	1	2.2 (0.1–11.8)	0	0.0 (0.0–2.4)
Depressed	0	0.0 (0.0–7.9)	0	0.0 (0.0–2.4)
Other problem	4	8.9 (2.5–21.2)	28	18.1 (12.4–25.0)
Other acute Illness	4	8.9 (2.5–21.2)	29	18.7 (12.9–25.8)

Appendix Table 3. Estimates of the number and percentage of individuals experiencing each symptom by sex, with 95% binomial CIs, among the confirmed dengue cases

Symptom	Male patients, n = 115	% (95% CI)	Female patients, n = 85	% (95% CI)
Alopecia	1	0.9 (0.02–4.8)	24	28.2 (19.0–39.0)
Tiredness	10	8.7 (4.3–15.4)	7	8.2 (3.4–16.2)
Resumed daily activities	115	100.0 (96.8–100.0)	85	100.0 (95.8–100.0)
Headaches	2	1.6 (0.3–6.3)	4	4.7 (1.3–11.6)
Muscle pain	2	1.6 (0.2–5.9)	1	1.2 (0.03–6.4)
Joint pain	0	0.0 (0.0–3.2)	3	3.5 (0.7–9.9)
Loss of appetite	2	1.6 (0.3–6.3)	1	1.2 (0.03–6.4)
Blurred vision	15	13.0 (7.5–20.6)	7	8.2 (3.4–16.2)
Rash	15	13.0 (7.5–20.6)	6	7.0 (2.6–14.7)
Sleep problem	7	6.1 (2.5–12.1)	2	2.3 (0.3–7.9)
Concentration problem	8	7.0 (3.1–13.2)	11	12.9 (6.6–22)
Little interest	1	0.9 (0.02–4.8)	0	0.0 (0.0–4.2)
Depressed	0	0.0 (0.0–3.2)	0	0.0 (0.0–4.2)
Other problem	8	7.0 (3.1–13.2)	24	28.2 (19.0–39.0)
Other acute Illness	19	16.5 (10.3–24.6)	14	16.5 (9.3–26.1)

Appendix Table 4. Logistic regression results for the outcome of experiencing each symptoms vs not experiencing it in the follow up period among dengue cases

Symptom	Variable	Coeff estimate	p value
Alopecia	Intercept	-6.46	<0.001
	Gender	3.76	<0.001
	DENV 2	0.42	0.7
	DENV 3	-14.8	0.99
	DENV 4	0.89	0.10
	(DENV1 reference group)		
	Probable primary	0.38	0.7
	Probable secondary	0.54	0.5
	(Indeterminate reference group)		
	Severity 2	0.66	0.34
	Severity 3	-15.6	0.99
	(Severity 1 reference group)		
	Age	1.01	0.2
Tiredness	Intercept	-2.7	<0.001
	Gender	-0.11	0.8
	DENV 2	-17	0.99
	DENV 3	1.1	0.35
	DENV 4	0.55	0.32
	(DENV1 reference group)		
	Probable primary	-17	0.99
	Probable secondary	-0.3	0.64
	(Indeterminate reference group)		
	Severity 2	-0.29	0.7
	Severity 3	-17	0.9
	(Severity 1 reference group)		
	Age	0.96	0.23
Headaches	Intercept	-19	0.99
	Gender	1.16	0.22
	DENV 2	-17	0.99
	DENV 3	-17	0.96
	DENV 4	0.65	0.47
	(DENV1 reference group)		
	Probable primary	-0.006	1
	Probable secondary	17	0.99
	(Indeterminate reference group)		
	Severity 2	-0.5	0.65
	Severity 3	-17	0.99
	(Severity 1 reference group)		
	Age	-1.5	0.09
Muscle pain	Intercept	-19	0.99
	Gender	-0.99	0.44
	DENV 2	-19	0.99
	DENV 3	-19	0.99
	DENV 4	-19	0.99

Symptom	Variable	Coeff estimate	p value
	(DENV1 reference group)		
	Probable primary	-20	0.99
	Probable secondary	-2	0.08
	(Indeterminate reference group)		
	Severity 2	-17	0.99
	Severity 3	-17	0.99
	(Severity 1 reference group)		
	Age	17	0.99
Joint pain	Intercept	-39	0.99
	Gender	19	0.99
	DENV 2	-15	0.99
	DENV 3	-18	0.99
	DENV 4	0.9	0.49
	(DENV1 reference group)		
	Probable primary	0.36	1
	Probable secondary	0.99	0.99
	(Indeterminate reference group)		
	Severity 2	0.4	1
	Severity 3	-17	0.99
	(Severity 1 reference group)		
	Age	-2.5	0.053
Loss of appetite	Intercept	-39	0.99
	Gender	-0.7	0.64
	DENV 2	-17	0.9
	DENV 3	3	0.0323
	DENV 4	1	0.54
	(DENV1 reference group)		
	Probable primary	18	0.9
	Probable secondary	17	0.9
	(Indeterminate reference group)		
	Severity 2	-16	0.9
	Severity 3	-17	0.9
	(Severity 1 reference group)		
	Age	17	0.9
Blurred vision	Intercept	-3.6	0.005
	Gender	-0.35	0.49
	DENV 2	-0.24	0.8
	DENV 3	1.9	0.06
	DENV 4	0.05	0.9
	(DENV1 reference group)		
	Probable primary	1.4	0.24
	Probable secondary	1.2	0.27
	(Indeterminate reference group)		
	Severity 2	-0.41	0.6
	Severity 3	-14	0.99
	(Severity 1 reference group)		
	Age	0.52	0.45
Other problem	Intercept	-0.9	0.12
	Gender	0.72	0.02
	DENV 2	-0.5	0.65
	DENV 3	0.6	0.9
	DENV 4	0.2	0.34
	(DENV1 reference group)		
	Probable primary	0.08	0.6
	Probable secondary	0.18	0.49
	(Indeterminate reference group)		
	Severity 2	-0.2	0.65
	Severity 3	-16	0.99
	(Severity 1 reference group)		
	Age	0.34	0.38

Appendix Table 5. Logistic regression results for risk of experiencing the symptoms post- acute with the predictor variable of whether this experience was experienced in the acute phase. This is only done for those symptoms that were recorded in the acute and follow-up period.

Outcome	Coefficient and p-value
Muscle pain post-acute	Intercept -2.0396 1.66e-14 ***; retro_pain_acute -0.1760 0.728
Blurred vision post-acute	(Intercept) -3.2581 .06e-08 ***; muscle_pain_acute -18.3080 0.995
Joint pain post-acute	(Intercept) -21.57 0.994; joint_pain_acute 17.97 0.995

Appendix Table 6. Occurrence of symptoms, if reported, within the defined time periods after the final acute illness assessment

Symptom	<7 days	8-30 days	31-90 days	Unknown	Total
Tiredness	16	1	0	0	17
Headaches	5	0	1	0	6
Muscle pain	2	1	0	0	3
Joint pain	2	0	1	0	3
Loss of appetite	3	0	0	0	3
Blurred vision	2	2	16	2	22
Rash	15	5	1	0	21
Sleep problems	4	1	2	2	9
Concentration problems	2	1	5	11	19
Little interest	0	1	0	0	1
Other problems	6	3	23	0	32
